# Enhancing theoretical BLS knowledge with virtual reality: a randomized controlled trial in medical students

**DOI:** 10.1016/j.resplu.2025.101169

**Published:** 2025-11-19

**Authors:** Nico Tannemann, Olga Tsarenko, Frank Herbstreit, Margarita Gestmann, Thorsten Brenner, Cynthia Szalai

**Affiliations:** a Department of Anesthesiology and Intensive Care Medicine, University Hospital Essen, University Duisburg-Essen, Essen, Germany; b Faculty of Medicine, University Duisburg-Essen, Essen, Germany

**Keywords:** Medical training, Resuscitation training, Digital methods, Digital education, Simulation training

## Abstract

**Background:**

High-quality cardiopulmonary resuscitation (CPR) training, including both technical and non-technical skills, is essential for medical students. Virtual reality (VR) offers immersive learning environments that may enhance traditional teaching methods. This study investigates the impact of a single VR session prior to an Advanced Life Support (ALS) course on knowledge and performance of basic life support skills among medical students.

**Methods:**

In this single blind randomized controlled trial, 126 fourth-year medical students with prior Basic Life Support (BLS) training were assigned to either an intervention group (*n* = 66) with an additional 3-part immersive VR session covering BLS theory and practice or a control group (*n* = 60) receiving standard preparation. All participants underwent a seminar based on advanced life support principles as dictated by the European Resuscitation Council (ERC) and International Liaison Committee on Resuscitation (ILCOR) guidelines. Theoretical knowledge was assessed via multiple-choice questionnaires at three time points (baseline, post-course, 12 weeks later). Practical skills were evaluated through an Objective Structured Clinical Examination (OSCE). Data were analyzed using Wilcoxon tests, repeated-measures ANOVA, and linear mixed models. Student evaluations were used to gauge subjective satisfaction with the scenario during teaching,

**Results:**

No significant differences were observed between groups at baseline. The intervention group demonstrated significantly greater gains in knowledge at both post-course (*p* < 0.01) and follow-up (*p* = 0.04). However, no significant differences were found in OSCE performance. The VR group’s improvement over time was significantly higher, suggesting a positive effect of VR on knowledge retention. Students were satisfied with the addition of a VR scenario in the teaching format.

**Conclusion:**

A single VR session prior to ALS training enhanced theoretical knowledge but did not significantly affect practical performance. Students were open to integration of the technology into training, so that VR may serve as a valuable adjunct in CPR education. Further research is needed to evaluate its long-term impact and the optimal integration method into curricula.

## Introduction

### Background and context

Cardio-pulmonary resuscitation (CPR) including basic life support (BLS) as well as advanced life support (ALS) are crucial skills for every healthcare provider.^1^ Therefore, effective CPR training should be a high priority learning objective in medical training. BLS is an essential building block for ALS skills and in fact the key components of BLS such as early recognition of cardiac arrest, effective chest compressions, timely use of a defibrillator and early onset call for expert help are associated with positive outcomes regarding survival.^2^ The outcome of CPR is further dependent on whether the initial rhythm was shockable or not.^3^ The delay to defibrillation is inversely correlated with the outcome of shockable rhythms, especially ventricular fibrillation^4^ highlighting the importance of early defibrillation. As stated by the European Resuscitation Council (ERC), BLS should be taught using scenario-based simulation methods using resuscitation manikins with feedback function. Furthermore, the ERC suggests a team-based training approach,^5^ allowing learners to repeatedly practice emergency protocols and rare scenarios without risking patient safety.^6^

### Rationale and summary of existing evidence

In recent years, there has been a growing availability of digital learning formats.^7^

Virtual reality (VR) has an increasing relevance in our daily lives. There are many potential uses in medical education.^7^ VR enables an immersive experience that creates a strong sense of presence, giving users the impression of actually being in the simulated environment.^8^ This enhanced sense of reality goes beyond what traditional simulations can offer, potentially making VR particularly suitable for use in educational and training contexts. This is especially important in the context of experiential learning. This concept emphasizes the role of direct, hands-on experience and the cyclical process of concrete experience and abstract conceptualization.^9^ VR enables the students to reflect on experiences and revisit their activities within the virtual environment.^10^ Furthermore, students take an active role in constructing new knowledge and developing skills, integrating these with their existing understanding and prior experiences in line with the concept of constructivism.^11^ Previous studies in lay populations have demonstrated that VR-based training can lead to improvements in manual resuscitation skills, particularly chest compressions, as well as increased self-efficacy.^12^ Virtual reality also offers the advantage of being instructor independent, reduced personnel and financial requirements and multiple/repeated use as opposed to simulation training.^13^

### Research question and aims

Since there is lack of evidence regarding the effectiveness of VR assisted training for BLS training in medical students, we hypothesize that VR training will improve theoretical BLS knowledge and potentially practical skills in ALS simulation settings. To research if VR training will have the assumed effects, we conducted a randomized controlled trial with the intervention group undergoing a single session of VR BLS training prior to traditional training of ALS skills.

## Material and methods

### Study population

We conducted an explorative study with a single blind component and a content analysis of student evaluation to investigate the benefit of VR supported BLS training in medical students. The study collective consists of fourth clinical semester medical students with varying previous experience in BLS skills. All students enrolled in the Block Practicum Emergency Medicine of the Medical Faculty of the University Duisburg-Essen from 2023 (*n* = 186) were invited to participate in this study. Two students declined participation. The remaining students were randomly assigned to either the intervention group (*n* = 89) or the control group (*n* = 95) based on their course enrolment time. We only regarded participants who completed all three questionnaires (*n* = 126, control group *n* = 60, intervention group *n* = 66). The raters were unaware of the student group (control or intervention) affiliation. Therefore, the study was single blinded.

Verbal and written informed consent were obtained from all participants. Data was anonymized, and all possible identifying markers were removed. This study was approved by the Ethics committee of the Faculty of Medicine of the University Duisburg-Essen (**23**-**11318-BO**). The work has been carried out in accordance with the Declaration of Helsinki.

### Course concept

Both groups underwent a course based on ALS principles consisting of four sessions in a two-week course as part of the Block Practicum Emergency Medicine at the Medical Faculty of the University of Duisburg-Essen.^14^ The first lecture included a theoretical introduction to and an overview of the current ALS algorithm to ensure a common basic knowledge. In addition, a refresher of the BLS skills as well as an introduction of the required equipment took place. In the following three lessons, we focused on practical training of ALS skills, we put emphasis on algorithm-based chest compressions, effective and safe manual ventilation, the proper and safe use of a defibrillator, and the intravenous drug administration. Practical training based on ALS principles was performed by teams of three students each assigned a specific role of either team leader (TL), team member one and member two (TM1/TM2). For details of each role please see Appendix.

### Virtual reality training

The intervention group additionally completed a VR hospital based BLS scenario (ViREED®). The scenario consisted of three parts each approximately 5–10 min long. Students completed a BLS theory training, an interactive feedback-based scenario to practice efficient cardiac compressions, including depth, frequency and timing of compressions. They then completed a virtual resuscitation scenario requiring recognition of an unconscious patient, initiating chest compressions, requesting and using an AED. The VR scenarios require the Meta quest 3 headsets and are completely headset based requiring two hand controllers but no additional laptops. Voice command possibility was enabled.

### Evaluation of knowledge and skills

**Fig. 1:**
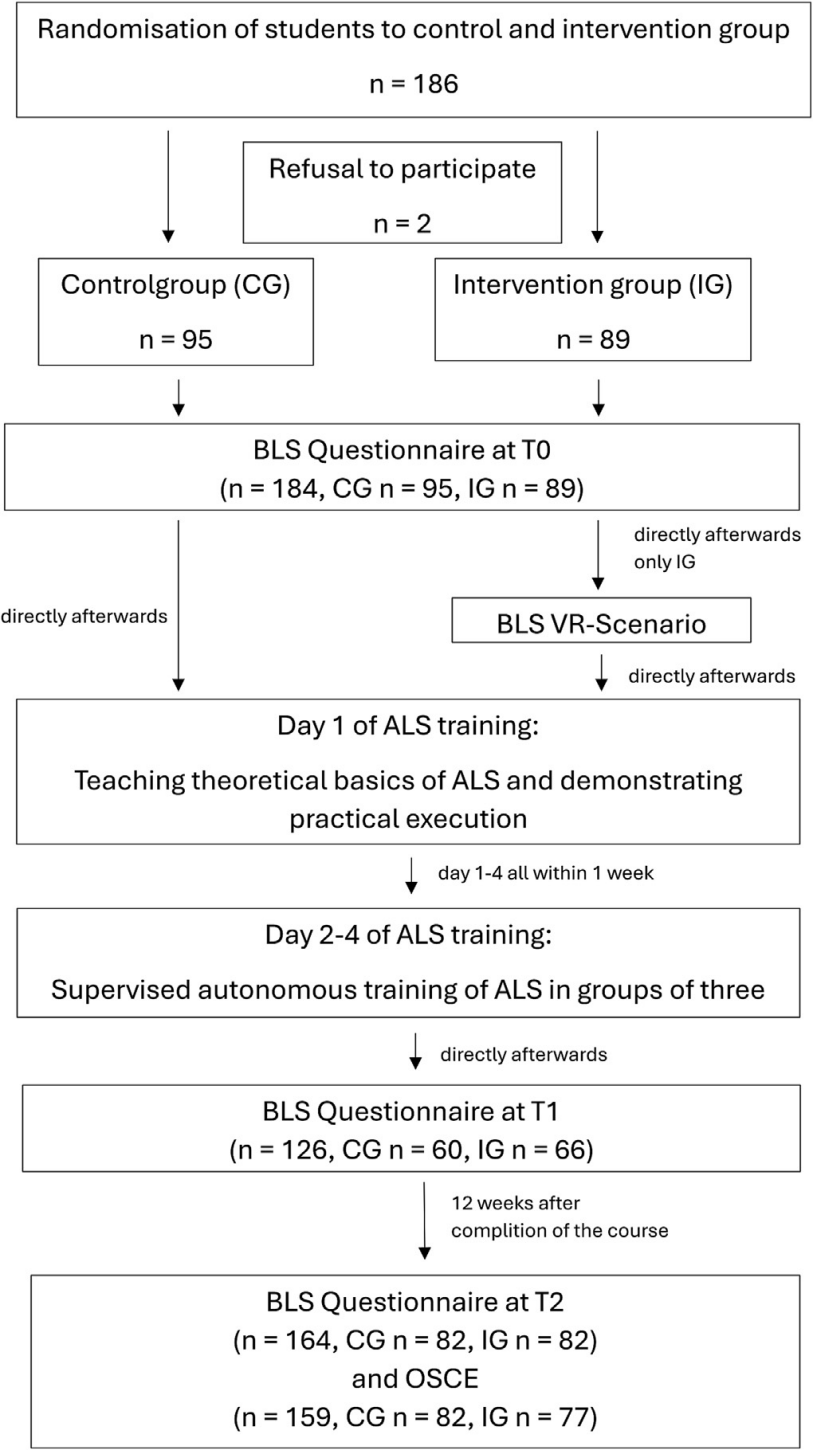
**Course timeline and study design. The figure illustrates the course timeline and the number of participants in the control and intervention groups at the respective time points**.

We assessed theoretical knowledge on BLS principles by using a 10-item multiple-choice questionnaire before, directly after and 12 weeks after (an 11-item questionnaire) the seminar based on ALS. These questionnaires were developed based on learning objectives recommended by the faculty’s emergency medicine curriculum. Details about the study design are provided in Fig. 1.

After the course, all students completed an OSCE station on ALS principles. The role (TL, TM 1, TM2) was randomly assigned to the students and a standardized 10 item checklist was used to evaluate performance. The ten item OSCE checklist was based on the European Resuscitation Council and International Liaison Committee on Resuscitation (ILCOR) guidelines and have not been independently validated. BLS skills such as recognition of an arrested patient, cardiac compressions (depth, frequency and effectiveness), timing and positioning and use of defibrillator were also assessed. Competencies were graded on a scale of 0–2 points “completely accomplished” (2 points) if the requirements were met entirely, “partly accomplished” (1 point) for minor mistakes and “not accomplished” (0 points) if the requirements were not met at all.

### Statistical methods

Descriptive characteristics such as median age, gender, prior BLS/ALS skills were recorded. Wilcoxon tests were performed to assess differences in age and a chi-square-tests for distribution of gender and prior medical experience between the control and the intervention group to determine structural differences between the two groups.

To estimate the influence of VR training on the student’s ALS competencies, we first calculated the median scores for each questionnaire and the OSCE station. As the third questionnaire consisted of 11 questions as opposed to the other questionnaires, which consisted of 10 questions, we further aligned the score of the third questionnaire by dividing it by 11 and multiplying it by 10. Wilcoxon tests were used to compare the results of the first, second and third questionnaire between the two groups. To further investigate the group differences across time points, we conducted a repeated-measures ANOVA with the different timepoints as a within subject factor and group (intervention vs. control) as a between-subjects factor using the R package ez.^15^ To account for individual variability and better quantify the interaction between time and group while accounting for individual-level variation, we additionally performed a linear mixed model (LMM) with group, time, and their interaction as fixed effects, and random intercepts for participants. To assess the variance explained by the fixed effects, we used Marginal and Conditional *R*^2^.^16^ As a measure of effect, we calculated the *η*^2^_partial_ with the commonly used cut-offs for small (*η*^2^_partial_ ≈ 0.01), medium (*η*^2^_partial_ ≈ 0.06) and large (*η*^2^_partial_ ≈ 0.14) effects^17^ for the ANOVA and LMM.

The statistical analyzes were performed using the R software^18^ with the ez package for the ANOVA analysis.^15^ LMM was performed using the R packages lmerTest^19^ and lme4.^20^ The figures were plotted using ggplot2.^21^ The marginal and conditional *R*^2^ as well as the *η*^2^_partial_ for the LMM were calculated using the effect size package.

### Student evaluation

The evaluation of individual courses by students is carried out by the faculty at the end of each semester and provides a subjective overview of the learning and teaching activities from the students themselves. These results were used to compare student responses for the emergency medicine practicum regarding the implementation and use of the VR BLS scenario during the resuscitation training courses. A total of 70 students (students from the intervention group) were eligible for comments, and 48 responses of varying lengths were obtained. All comments obtained were anonymous, no identifying markers were possible. The comments were collated and coded using a thematic approach thus multiple themes were sometimes generated from single comments.^22^, ^23^ Two authors participated in the manual coding process to maintain evaluative rigor in data analysis.

## Results

### Sample characteristics

Table 1 shows descriptive data of the study population. The reported previous medical experience included varying levels of paramedic, medical laboratory technician, radiology technician, operating room technician, nursing and midwifery and physiotherapy experience. There were no significant differences regarding age, gender distribution, or prior medical experience between the intervention and control group.

**Table 1:** Sample characteristics at baseline.

	**Total** ***N* = 126**	**Intervention** ***N* = 66**	**Control** ***N* = 60**	***p*-value**	**Missing**
Age*	24 (23–25)	24 (23–25)	24 (23–24,3)	0.53	5 (4 %)
Female+	79 (62.7 %)	44 (66.7 %)	35 (58.3 %)	0.43	0
Male+	47 (37.3 %)	22 (33.3 %)	25 (41.7 %)		
Medical Training+	34 (27 %)	20 (30.3 %)	14 (23.3 %)	0.50	0

Demographics at baseline, data given as * median (Q1–Q3) +*n*, *p*-Value for intervention group compared with control group, medical training is defined as any form of medical education prior to begin of medical school.

* *t*-test.

+ chi square test.

### Performance in a questionnaire and OSCE station

**Fig. 2:**
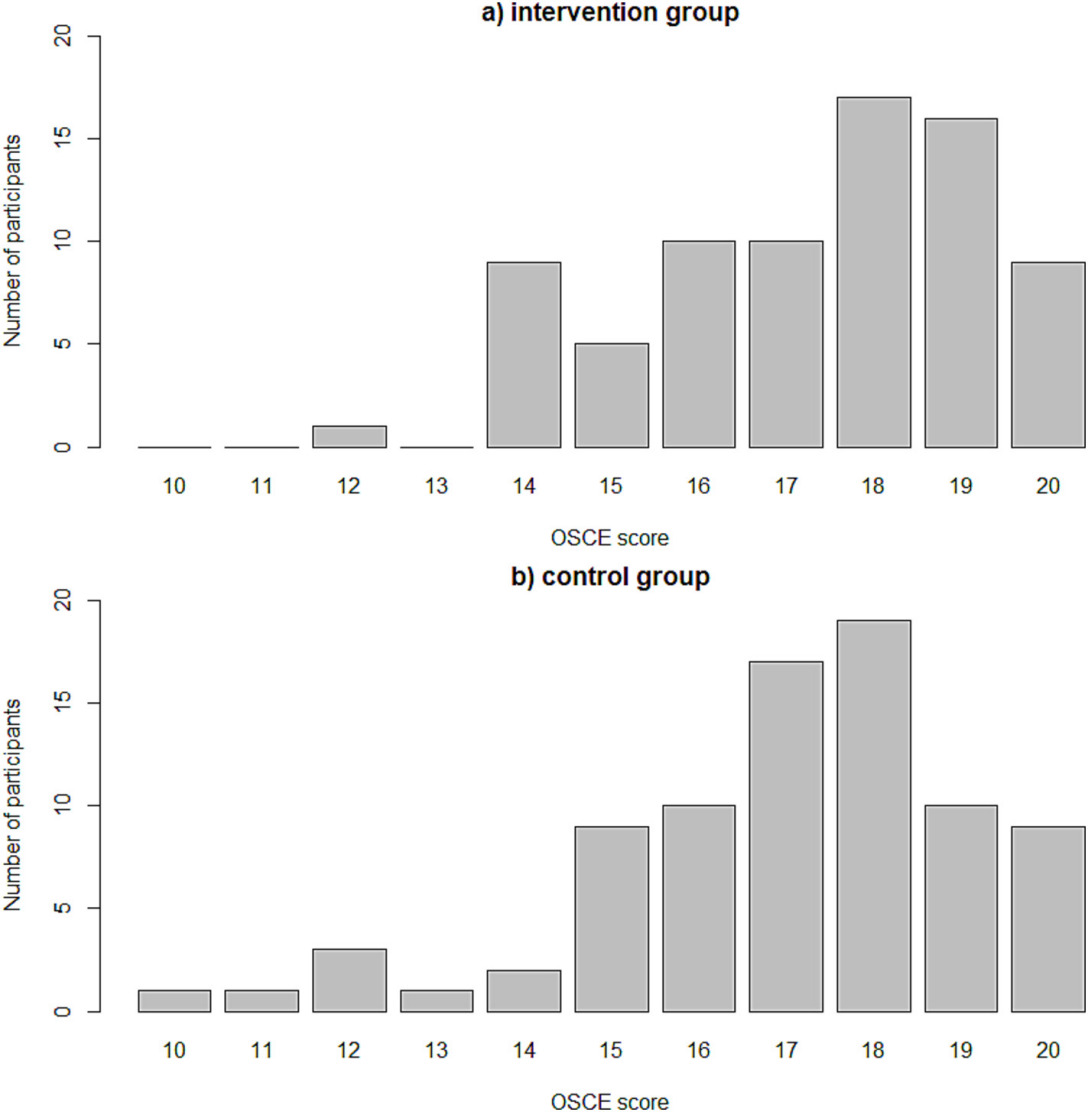
**Distribution of students’ scores in OSCE. (a) For the intervention group and (b) for the control group**.

Comparison of results between the intervention and control group (see Table 2) showed no significant difference in the baseline questionnaire T0 However, although there was improvement in the median score directly after the CPR training (two weeks later) in both groups (T1), the median score in the intervention group was significantly higher. Similar results were also consistent up to 12 weeks after the CPR (T2) course with the intervention group maintaining a significantly higher score than the control group. No significant difference was seen in practical skills at the OSCEs between the groups. Fig. 2 illustrates the distribution of students’ scores in the control and intervention group.

**Table 2:** Median scores by group and time points.

	**Total** ***N* = 126**	**Intervention** ***N* = 66**	**Control** ***N* = 60**	***p*-value**
T0	7 (7–8)	7.5 (7–8) ± 1.31	7 (7–8)	0.73
T1	8 (8–10)	9 (8–10)	8 (7.8–9)	<0.01
T2	9.1 (8.2–10)	10 (9–10)	9.1 (8.2–10)	0.02
OSCE	18 (16–19)	18 (16–19)	17 (16–18)	0.40

Data presented as median (Q1–Q3).

*p*-values given as Wilcoxon tests between intervention and control group.

T0: Score at baseline (before VR intervention).

T1: Score at first timepoint (2 weeks after VR intervention).

T2: Score at second timepoint (12 weeks after VR intervention).

The ANOVA analysis showed an overall difference in the groups mean scores (*η*^2^_partial_ = 0.037, *p* < 0.01), moreover both groups showed a change in their mean scores at the different timepoints (*η*^2^_partial_ = 0.276, *p* < 0.01). The ANOVA suggested that the mean scores of both groups evolved differently (*η*^2^_partial_ = 0.029, *p* < 0.01), considering the median scores shown in Table 2, indicating a higher improvement in the intervention group. The Mauchly’s test for sphericity was not significant (*p* = 0.57), therefore we used uncorrected *F*-values (Table 3).

**Table 3:** Results of repeated-measures ANOVA.

	***F***	**df**	***η*^2^_partial_**	***p*-value**
Group	13.27	1, 124	0.037	<0.01
Timepoint	74.22	2, 248	0.276	<0.01
Group × Timepoint	5.82	2, 248	0.029	<0.01

ANOVA test results; *F*: ANOVA test statistics; df: degrees of freedom.

**Fig. 3:**
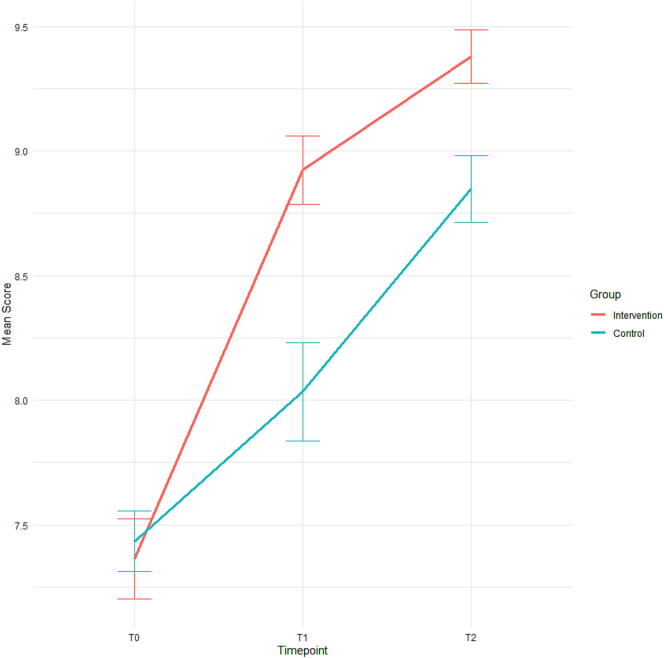
**Mean scores of the intervention and control group at T0, T1, and T2. T0: Score at baseline, T1: Score after VR intervention, T2: Score 12 weeks after VR intervention, error bars: standard deviation**.

In line with the ANOVA the results of the LMM (linear mixed model) revealed significant effects for interaction between group and timepoint (see Fig. 3). While there was no significant main effect of the group (control vs. intervention) at baseline, the mean score of the intervention group improved at T1 and T2 compared to baseline. However, significant interaction between group and timepoint indicated that the intervention group improved more over time compared to the control group. Specifically, the intervention group had significantly higher increases from baseline to T1 (*p* < 0.01) and T2 (*p* = 0.04). 31 % of the variance were explained by fixed effects (*R*^2^*m* = 0.31) whereas 34 % of the variance were explained by fixed and random effects (*R*^2^*c* = 0.34). The *η*^2^_partial_ for interaction between group and timepoint was 0.04. See Table 4 for detailed model estimates.

**Table 4:** Results of the linear mixed model.

	**Difference in mean score** **±se**	***η*^2^_partial_**	***p*-value**
Control Group vs. Intervention Group at baseline	−0.07 ± 0.15	0.10	0.73
Intervention Group T0 vs T1	1.56 ± 0.2	0.37	<0.01
Intervention Group T0 vs T2	2.02 ± 0.2		<0.01
Control Group vs Intervention Group from T0 to T1	0.91 ± 0.28	0.04	<0.01
control group vs intervention group from T0 to T2	0.60 ± 0.28		0.04

Data presented as difference in mean score for the mentioned groups respectively timepoint; se: standard error.

*η*^2^_partial_ given for groups, timepoint and interaction between group and timepoint.

*R*^2^*m* = 0.34, *R*^2^*c* = 0.31.

### Satisfaction with VR Module

Based on a content analysis of data obtained from the evaluation comments, two general themes were generated. From these central themes, subthemes were established, which illustrated and supported the main topics, see Table 5.

**Table 5:** Results of student evaluation.

**Theme**	**Subtheme**
Satisfaction with the VR Module (36)	Acceptable learning method (9) Complementary addition to seminars (5) Fun factor (23) Repeatability
Dissatisfaction with VR Module (12)	Technical challenges (8) Skepticism on advantageous effect of VR in teaching and learning (4)

Thirty-six from the 48 comments reported positive feedback to integration of the VR scenario in the courses. Responses mainly indicated that students had “*fun”* during the scenarios, or that the scenario was “*supercool”*. Most comments embraced the addition of the VR in the practicum as an acceptable learning format which allowed repeatability as well as providing a level of security when making mistakes, “ *I liked that I didn’t have to look silly in front of my colleagues like in the simulation”* or comments such as “*it would be great to get to practice the scenario at home”* support these subthemes.

Dissatisfaction with VR Module was generally limited to technical difficulties that students experienced and was reported in 12 comments. Comments were based on technical challenges experienced during the scenario relating to difficulty with the voice recognition, scenario crashes after one update as well as difficulty in spatial orientation.

## Discussion

No significant differences were observed between the control and intervention groups regarding demographics and previous medical experience. This suggests that the two groups were homogenous. This is particularly relevant, as such factors are known to influence the acceptance and effectiveness of technology and therefore VR-based interventions.^24^ Moreover, there were no significant differences between the groups in terms of baseline median scores prior to the intervention, suggesting a comparable baseline level of knowledge or performance.

The ANOVA revealed that the mean scores of both groups changed differently over time. The statistical significance indicates meaningful differences in how the groups developed, suggesting that the intervention of the VR scenario had a measurable and differentiated impact on knowledge acquisition and retention up to 12 weeks later. The results of the further conducted LMM support these findings. There were no significant differences between the control and intervention group at baseline. However, significant interactions between group and timepoint indicated that the knowledge of the control group improved less over time compared to the intervention group. Specifically, the control group had significantly lower increases from baseline to T1 (*p* < 0.01) and T2 (*p* = 0.036) than the intervention group. The *η*^2^_partial_ for interaction between group and timepoint of 0.029 in the ANOVA and 0.04 in the LMM suggests a small to medium effect of those interactions on the endpoint.^17^ Notably, the difference between the groups was more pronounced from baseline to T1 than from baseline to T2, suggesting that the intervention had its strongest impact in the immediate post-intervention phase, with a slight convergence of group performance over time. This is not surprising, given that the VR intervention took place only once. Repeated or sustained interventions might lead to improved learning retention effects. Suggesting repeated confrontation within a VR Scenario may increase the retention of theoretical knowledge.^25^

The variance explained by fixed effects (*R*^2^*m* = 0.31) and by fixed and random effects (*R*^2^*c* = 0.34) indicates a moderate to high model fit.^16^

No significant differences were found between the groups in the OSCE results. This may reflect the limited exposure to the VR intervention, as performance improvements often require repeated practice. Supporting this interpretation, Bartlett et al.^26^ observed a noticeable increase in the learning curve for surgical VR training only after the third session, suggesting that a single exposure may be insufficient to produce measurable gains in applied performance. Although no significant difference was seen in the overall scores between the two groups, more students in the intervention group (54.55 %) scored at least 18 points than in the control group (46.34 %). This observation, however, may be due to other factors, as BLS skills were assessed in the context of an ALS scenario and may not be directly attributed to improved BLS skills. However, gaining theoretical knowledge is the first step of procedural learning and helps build a cognitive model of the task.^27^

Although we did not see any improvement of practical skills in our setting, we could prove an improvement in theoretical knowledge as a foundation for procedural learning. Repeated exposure to the VR scenario and revaluation regarding long-term persistence of the improvement and possible improvement of practical skills seems to be meaningful extrapolation. However, a differentiated evaluation of individual skills such as defibrillator use or chest compressions was not possible due to the study design, a more differentiated view could be meaningful in further research. Furthermore, we suggest that VR training could be used as an addition to our conventional course concept. VR application in other learning contexts may be useful, as there is evidence that even laypersons could profit from VR based BLS training.^12^

Students reported general acceptance and satisfaction with the integration of a VR scenario into emergency medicine practicum. Acceptance of digital technologies in teaching isn’t a new phenomenon, literature suggests that VR is increasingly being accepted and validated in the teaching context^28^ with multiple advantages such as repeatability, cost effectiveness and increased learning retention. New technologies offer multiple scenarios which promote interprofessional learning, teamwork and shared decision-making^29^ which are all required skills for effective CPR. Despite the overall satisfaction with VR as a teaching adjunct and the significantly improved theoretical knowledge, the dissatisfaction with this technology in the learning setting cannot be ignored. The typical student was about 24 years of age suggesting that were in the digital native generation.^30^ However, the comments show that some students were challenged with the technology. This result is also not specific to our cohort, some literature suggests that acceptance of digital tools is not specific to age, status or experience.^31^ The acceptance of these in learning settings may also be influenced by attitudes and skill-levels of lecturers.^32^ This especially highlights that even considering the increasing introduction of digital tools such as VR in teaching and learning, traditional concepts which target various types of learning styles must also be retained, as students employ various learning styles which should all be accommodated.^33^

Since the scenarios are adaptable, based on guidelines for different professions and situations, the VR scenario is not exclusively restricted to the training of medical students, but also suitable for training of different healthcare professions such as nursing and paramedics. However, the VR-scenarios should be adapted to achieve learning objectives for these professions. For example, in nursing staff, ward-based scenarios could be developed that reflect typical in-hospital emergencies, focusing on the early recognition of patient deterioration, activation of the resuscitation team, and initiation of BLS until further assistance arrives. In prehospital settings such as for paramedics, the VR scenarios could realistically simulate an out of hospital setting with teamwork in small crews. Besides the integration of VR-scenarios in BLS training in the form of multi-session approaches, the use of VR-scenarios as an additional self-learning tool offers the advantage of repeatability and the flexibility of remote learning. The VR scenario, due to its versatility, can also be used in multiple teaching settings such as pre-course preparation for seminars based on ALS principles or integrated into a flipped classroom teaching concept. Since the concept underscores the significance of learner autonomy or agency and individualized learning pathways, allowing students to take ownership of their educational experience in line with the constructivist learning theory^34^ and gives the students the autonomy to deepen their knowledge.

### Limitations

This study has several methodological strengths. It was designed as a randomized controlled trial. Furthermore, there were no structural differences between the control and intervention groups regarding key variables. The longitudinal design allowed for the observation of changes over multiple time points, providing insight into the development of learning effects over time. However, certain limitations must also be acknowledged. The VR intervention consisted of a single session, which may have limited its long-term impact. Moreover, the final assessment point coincided with a high-stakes examination, which likely prompted increased preparation across both groups and may have confounded the intervention effect. In this context, especially regarding the overall high scores attained by the students, this might lead to a ceiling effect and complicate a nuanced evaluation of the VR-intervention’s effect. Furthermore, a possible explanation for the lack of significance in the OSCE score may be that BLS skills were incorporated into an ALS OSCE station, students may have concentrated on incorporating these skills into the ALS competencies rather than focusing specifically on BLS skills. Due to the set-up of the OSCE stations, only one rater was assigned to the OSCEs. An assessment of inter-rater reliability was not possible. Additionally, no conclusions can be drawn regarding the effectiveness of the intervention in real-life resuscitation scenarios or its potential impact on patient outcomes.

## Conclusion

In this study, a single VR session led to a greater improvement in theoretical knowledge compared to conventional methods of learning BLS theory. However, we observed no significant differences in the attainment of practical skills as assessed by the OSCE station. The short-term impact on manual performance appears promising; growing evidence suggests that VR can be a valuable supplement to conventional theoretical resusctitatino training. Student reported mainly positive responses to the inclusion of VR in teaching formats. Our study suggests that incorporating a VR scenario into teaching BLS skills to healthcare professionals, particularly medical students, is beneficial. A multi-session group protocol with an interprofessional approach to resuscitation, as recommended by the ERC/ILCOR,^1^ is promising and warrants further investigation. Nevertheless, further studies are needed to determine the optimal frequency and integration of VR into training curricula. The effect of VR-based education on real-life performance and patient outcomes remains uncertain and warrants future investigation.

## Declaration of generative AI and AI-assisted technologies in the writing process

During the preparation of this work the authors used ChatGPT (GPT-5) to improve language and readability as well as translating data to English. After using this tool, the authors reviewed and edited the content as needed and take full responsibility for the content of the publication.

## CRediT authorship contribution statement

**Nico Tannemann:** Writing – original draft, Methodology, Formal analysis, Data curation. **Olga Tsarenko:** Writing – review & editing, Investigation, Data curation. **Frank Herbstreit:** Writing – review & editing, Resources, Methodology, Conceptualization. **Margarita Gestmann:** Writing – review & editing, Methodology, Data curation, Conceptualization. **Thorsten Brenner:** Writing – review & editing, Supervision, Resources, Conceptualization. **Cynthia Szalai:** Writing – original draft, Supervision, Resources, Methodology, Investigation, Formal analysis, Data curation, Conceptualization.

## Declaration of competing interest

The authors declare that they have no known competing financial interests or personal relationships that could have appeared to influence the work reported in this paper.
