# Supplementary material for: Enhancing theoretical BLS knowledge with virtual reality: a randomized controlled trial in medical students
Source: Resusc Plus. 2025 Nov 19;27:101169. doi: 10.1016/j.resplu.2025.101169 (PMC12719688; doi:10.1016/j.resplu.2025.101169)
Supplement: Supplementary Data 1 [file mmc1.pdf]

Checklist ALS (Advanced Life Support)

Role 1: Team Leader

No. | Item | Correctly Performed | Performed | Not Performed | 2 pts | 1 pt | 0 pts

1. Initial Assessment (talking to the patient, applying pain stimulus, inspection and clearing the airway, checking breathing [see, hear, feel])

2. Informing the team of the need for resuscitation (Clear communication of the patient's condition and necessary actions)

3. Bag-mask ventilation (Airway management: bag-mask ventilation 30:2, oxygen)

4. Rhythm analysis, indication for defibrillation (ECG I with assistant 1, clear coordination)

5. ECG II (Correct defibrillation)

6. Medication administration (Adrenaline 1 mg: 9 ml NaCl)

7. Airway protection (with LMA, laryngeal tube - proper use of airway device, capnometry)

8. Indication for medication administration (timing and dosage in line with algorithm)

Total Score:

Critical Skills:

[ ] Able to make sound clinical decisions

[ ] Situational awareness

[ ] Able to manage all tasks

[ ] Worked well in a team

Side Assistant 1

Time Frame: You have 6 minutes!

No. | Item | Correctly Performed | Performed | Not Performed | 2 pts | 1 pt | 0 pts

1. Defibrillator & Analysis (with team leader: announce readiness for analysis, ensure the patient is not touched, remind team members if necessary)

2. Performing Defibrillation (correct energy level per guidelines, safe defibrillation)

3. Establishing IV access

4. Drawing up and administering medication (on instruction from the team leader: Adrenaline 1mg:9ml NaCl)

5. After role switch: chest compressions - start immediately after indication, loud announcement of target frequency, depth, correct technique - emphasis on quality

6. Chest compressions: pressure point, frequency, depth, recoil

7. No pauses > 30 sec in chest compressions

8. Communication with team leader (counting compressions/cycles aloud, feedback on ventilation effectiveness)

Total Score:

Critical Skills:

[ ] Able to make sound clinical decisions

[ ] Situational awareness

[ ] Able to manage all tasks

[ ] Worked well in a team

Side Assistant 2

Time Frame: You have 6 minutes!

No. | Item | Correctly Performed | Performed | Not Performed | 2 pts | 1 pt | 0 pts

1. Chest compressions - immediate start after indication, loud announcement of frequency, depth, correct technique - focus on quality: pressure point, frequency, depth, recoil

2. Chest compressions: pressure point, frequency, depth, recoil

3. No pauses > 30 sec in chest compressions

4. Switch between side assistants after 2nd rhythm analysis

5. Defibrillator & Analysis (with team leader: announce readiness, ensure no manipulation of the patient, inform team if needed)

6. Performing Defibrillation (correct energy per guideline, safe defibrillation)

7. Communication with side assistant

8. Communication with team leader (counting compressions/cycles aloud, feedback on ventilation effectiveness)

Total Score:

Critical Skills:

[ ] Able to make sound clinical decisions

[ ] Situational awareness

[ ] Able to manage all tasks

[ ] Worked well in a team
